# The Extent to Which Policies Are Supporting Families to Improve Child Mental Health Outcomes in Victoria, Australia: A Policy Scoping Review

**DOI:** 10.1002/hpja.70040

**Published:** 2025-04-09

**Authors:** M. Stonnill, S. Gray, S. Woolfenden, S. Goldfeld

**Affiliations:** ^1^ Centre for Community Child Health Murdoch Children's Research Institute Melbourne Australia; ^2^ Department of Pediatrics University of Melbourne Melbourne Australia; ^3^ Community Paediatric Research Group, Sydney Medical School University of Sydney Sydney Australia; ^4^ Sydney Institute for Women, Children and Their Families Sydney Local Health District Sydney Australia

**Keywords:** child wellbeing, family support, health policy

## Abstract

**Introduction:**

Child mental health, both in terms of addressing difficulties and promoting competence, is foundational to optimal health, educational and vocational outcomes across the lifecourse. It is a key policy focus in Australia. This study aims to describe the current child mental health policy landscape in Victoria and at a federal level within Australia, to understand the extent to which mental health competence is targeted and families are currently leveraged as a key influence on child mental health outcomes.

**Methods:**

Policy specific websites and search engines were used to identify relevant policy documents for inclusion in a policy scoping review.

**Results:**

Twenty‐five policies were identified as relevant for inclusion in the review across health, education, social services and Aboriginal and Torres Strait Islander national peak bodies or government departments. Twenty policies targeted competence, to varying extents. Twelve policies specifically focused on supporting families to improve child mental health outcomes, and 11 policies acknowledged families as a key influence on children but did not provide specific examples of how to support families. Two policies had no mention of families.

**Conclusions:**

There is currently a range of policies, at a state and national level, that aim to prevent and treat mental health difficulties and promote mental health competence. So What? There is an opportunity to improve these policies by clearly defining competence and providing guidance on how to support families.

## 1 Introduction

**FIGURE 1:**
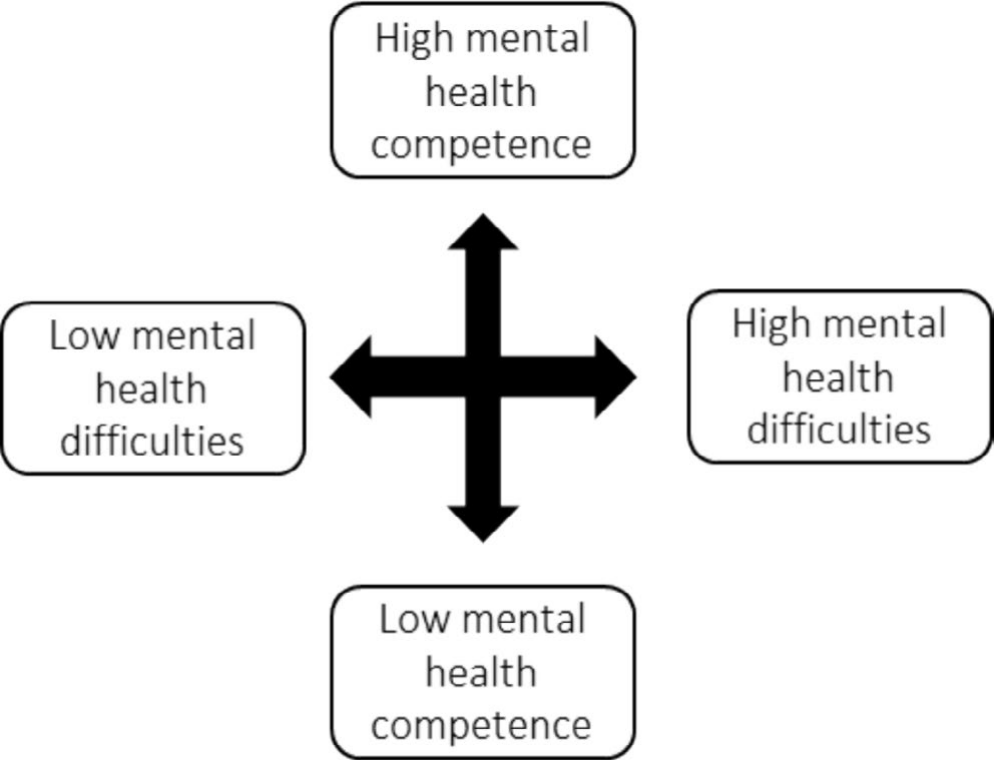
Dual mental health continuum (adapted from O'Connor et al. [9]).

Mental health exists on a dual continuum, which is comprised of mental health competence and mental health difficulties (Figure 1). Mental health competence is one conceptualisation of positive mental health, sometimes referred to as wellbeing, thriving or flourishing. Distinct from mental health difficulties, it is defined as success in areas such as social skills, emotional regulation and prosocial behaviours [1]. The development of competencies, such as social competence, and the experience of mental health difficulties during the early years have an enduring impact on learning, behaviour and health outcomes during childhood and into adolescence and adulthood [2, 3, 4, 5, 6]. Competence is positively linked with educational and employment outcomes in adolescence and adulthood [4]. Similarly, children with mental health difficulties, even those considered below the threshold for a diagnosis, are more likely to have a mental health disorder as an adult and struggle with other health, legal, financial and social outcomes [5, 7]. Competence is not fixed; it changes as children age and, like mental health difficulties, is influenced by the social determinants of health, with children from more advantaged backgrounds experiencing greater mental health competence [1]. Given the effect of child mental health on outcomes throughout the life course, there is an imperative to intervene early to improve the mental health of children (0–12 years) and protect them against future challenges as they transition to adolescence and adulthood. However, despite this, there has traditionally been a gap in policies and services targeting mental health competence to improve child outcomes (0–12 years) [8].

In response to this gap, there is increasing interest in child wellbeing and mental health policies internationally and within Australia. New Zealand and Australia have both developed child mental health and wellbeing policies to support mental health competence and prevent mental health difficulties in children [10, 11]; the OECD has developed a framework to measure child wellbeing to support this work more broadly [12]. These policies recognise that individuals experience the best outcomes if they are both free from mental health difficulties and experience high mental health competence [13]. A comprehensive child mental health policy agenda should target the promotion of mental health competence and the prevention of difficulties to ensure children experience the best start to life [14].

The increased policy interest in child mental health has translated into increased interventions to promote competence and prevent difficulties in Australia. In response to the Royal Commission into Victoria's Mental Health System, there has been unprecedented spending on child mental health, with the Victorian Government investing $217.8 million over 4 years from 2022, to support student mental health and wellbeing [8, 14]. Many of the policies and interventions targeting child mental health competence and difficulties focus on leveraging education settings, such as the South Australian Royal Commission into Early Childhood Education and Care [15], the Victorian Mental Health in Primary Schools program [16] and the Australian Student Wellbeing Framework [17]. However, as a central component of children's lives, families should also be considered when creating and implementing child mental health policies and interventions. The family environment is important for developing mental health competencies such as social–emotional skills, early academic skills and emotion regulation [18, 19, 20]. Similarly, family risk factors such as parent mental health difficulties and parenting behaviours are associated with mental health difficulties [21, 22, 23].

The investment in child mental health policy reflects the growing understanding that individual‐focused interventions must be complemented by public health approaches that promote mental health competence and prevent mental health difficulties [24]. An effective public health response requires a multisectoral approach that combines a range of interventions and policy approaches [24], because children's good mental health is shaped by the environments in which they live learn and develop [25]. In the context of families, this could include interventions that integrate mental health prevention and promotion into routine services, such as primary health care, home visiting, schools and community settings, could strengthen family environments, foster early resilience and reduce long‐term mental health disparities [24]. When considering where we would find these policies, we expect health departments to have a role in early detection and prevention through primary care and maternal child health services, education is central to school based initiatives and social services contribute through supporting families and early intervention services. As mental health is shaped by the environments we live in, urban planning and housing also play a role in influencing child mental health outcomes; however, we would not expect these departments to measure the mental health of children as key outcomes, where they do measure social outcomes, it is more likely to be those that affect adults. Given the strong policy focus on children's mental health, it is important to assess whether policies are adequately addressing the foundational role of families. There is a risk of overlooking a key setting for mental health promotion and prevention, limiting the effectiveness of interventions.

This policy scoping study aims to understand the extent to which Victoria and federal policies target mental health competence, and whether they have explicitly targeted families as a key influence on child mental health competence and difficulties for children aged 0–12 years. This is an important pulse check of the mental health policy landscape which has not previously been conducted; findings will help identify any gaps in the current policy response. This is essential to ensure that our policies not only address mental health difficulties but also promote mental health competence so that Australian children flourish. Australia has led the way in the design and implementation of mental health policies and services for young people aged 12–25 years, and while there are continued gaps in the mental health system for this group, these are outlined elsewhere and are not the focus of this study [26]. Considering the context of the Royal Commission into Victoria's Mental Health System and the large investment in student mental health initiatives, Victoria has been selected as an exemplar to allow us to look at the child mental health agenda nationally and how this is being implemented at a state level.

## 2 Methods

The policy scoping review was guided by the scoping framework developed by Arksey and O'Malley [26]. The scoping framework sets out four key stages.

### 2.1 Identify the Research Question

The policy scoping review aims to answer the following questions: (I) what current policy documents are being used in Victoria and nationally to inform work to improve child mental health outcomes (both by reducing difficulties and improving competence), (II) to what extent are infants and children targeted separately from adolescents and (III) to what extent do these policies support families to influence child mental health outcomes. It is not the aim to assess the quality of each policy document.

### 2.2 Identify Relevant Studies

To identify relevant policy documents, a broad search of the grey literature was conducted. Policy‐specific websites and search engines were used to identify relevant policy documents, including Australian Policy Online, Victorian Department of Health, Victorian Department of Education, Victorian Department of Families, Fairness and Housing, Australian Government Department of Education, Australian Government Department of Health and Australian Government Department of Social Services. These specific government departments were chosen as a focus as they were considered most likely to contain policy documents relevant to child mental health.

The search used keywords/phrases on government websites, including ‘child mental health’ and ‘child mental health and wellbeing’. Where available, topic filters were applied to narrow the search to relevant policies, a full list of search terms and filters can be found in Table A1.

Additionally, a snowball search strategy was used to source other relevant policies in the reference list of identified policy documents.

### 2.3 Study Selection

‘A policy is a set of ideas or plans that is used as a basis for making decisions’ [27], using this broad definition of policy, documents were included in the scoping review if they were considered a policy, plan or strategy that outlined a set of ideas intended to inform decision making or government direction around child mental health. Twenty‐five policies were identified as relevant for inclusion in the review. Table 1 outlines the inclusion and exclusion criteria used for study selection.

**TABLE 1:** Policy scoping inclusion and exclusion criteria.

Inclusion criteria	High level policy documents, strategic plans, key initiatives or frameworks that intend to inform decision making or government direction Published by the Victorian Government, the Australian Government or national peak body Current policy (implementation period includes 2024). Where there is no clear implementation period, must be reasonable to assume it influences current policy decisions, e.g., frameworks published in the last 10 years (2014–2024) Targets children 0–12 years Contains outcomes related to child mental health: ○ Child mental health difficulties—depression, anxiety, internalising behaviours, withdrawal, dysphoria, externalising behaviours, impulsivity, aggressiveness and disruptiveness ○ Child mental health competence—thriving, resilience, coping with stress and mental wellbeing. Policies did not have to only target children or mental health for inclusion, but they did need to have specific outcomes related to children and mental health.
Exclusion criteria	Documents targeting adolescents or adults with no reference to children 0–12 years Budgets, evaluation reports and evidence summaries Health practice guidelines

**FIGURE 2:**
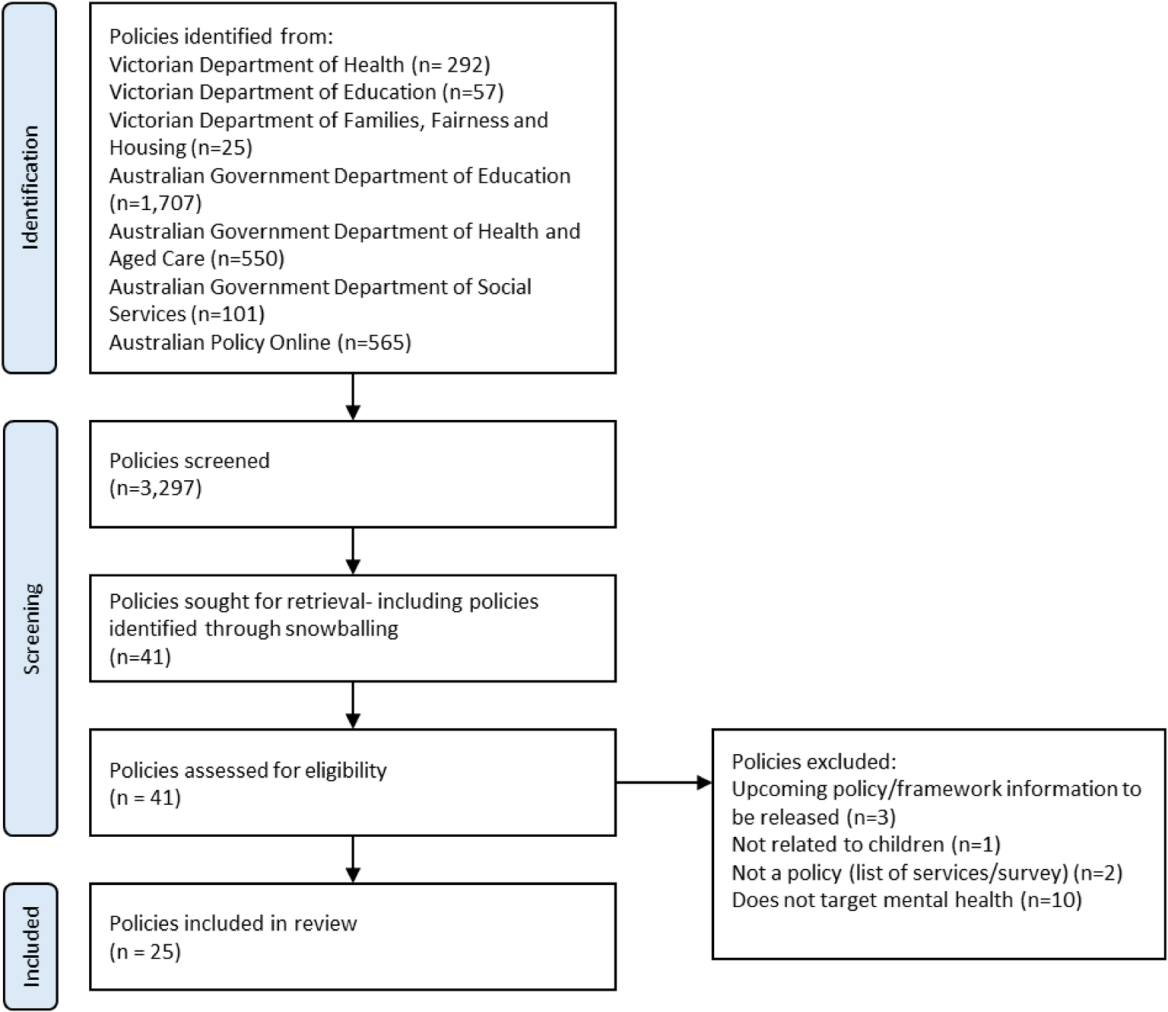
Policy selection flow diagram.

An initial screening based on title was conducted, 41 policies underwent full text screening, before the final 25 policies were selected for inclusion in the review. There was a low number of policies sought for retrieval compared to the number of policies screened as the grey literature search returned many irrelevant documents, for example, a search for child wellbeing on the Australian Government Department of Health and Aged Care also returned information relating to dental health, tobacco and the national redress scheme. Documents included websites with key information on policies, their aims and intended outcomes. Figure 1 provides an overview of the screening and policy selection process (Figure 2).

### 2.4 Charting the Data

Data was charted by recording key information into an excel document. Variables captured in the data extraction included key contextual information such as government department and date of publication, as well as outcome variables related to the research questions. The variables charted in excel are outlined in Table 2.

**TABLE 2:** Variables captured in the policy scoping data extraction.

Variable	Definition
Name	Document name.
Government department	Name of government department that published the document.
Date	Date of publication.
Overview	Brief overview of the aims and components of the policy or intervention.
Reference to child mental health	Document references child mental health outcomes or strategies to influence child mental health that are distinct from ‘child and adolescent’ mental health, recognising the differences in these age groups.
Policy targets mental health difficulties	Document aims and outcomes include preventing or reducing child mental health difficulties such as internalising (e.g., anxiety, withdrawal) and externalising (e.g., impulsivity, aggressiveness) problems.
Policy targets mental health competence	Document aims and outcomes include supporting the development of mental health competencies, including wellbeing and resilience. This is separate from the prevention of difficulties and may include measures of success in social competence, responsibility and respect, approaches to learning, readiness to explore new things and prosocial behaviours.
Policy leverages modifiable family variables	Document identifies families as a key influence on child mental health outcomes. This may include family variables such as parent mental health, parenting style, home learning and the home environment, family relationships, socioeconomic status and cultural determinants.

## 3 Results

Table 3 provides a summary of the 25 policies identified for inclusion in the scoping review. Eighteen federal and seven state‐level policies were included. Eleven relevant policies were identified from across health departments, seven from education departments, five from Aboriginal and Torres Strait Islander national peak bodies or government departments and two from social services.

**TABLE 3:** Policy source summarised by focus on difficulties, competence and extent of family supports.

Name	Department (date)	References child (0–12)	Targets difficulties	Targets competence	Supports families
Royal Commission into Victoria's Mental Health System [8]	Victorian Department of Health (2021)				
Victorian child and adolescent outcome framework [28]	Victorian Department of Health (2018)				
The Victorian early years learning and development framework [29]	Victorian Department of Health (2016)				
Framework for Improving Student Outcomes (FISO 2.0) [30]	Victorian Department of Education (2022)				
Mental Health Fund and Menu [14]	Victorian Department of Education (2022)				
Mental Health in Primary Schools [16]	Victorian Department of Education (2022)				
Australian Student Wellbeing Framework [17]	Australian Government Department of Education (2018)				
Be You‐ national education initiative for mental health [31]	Australian Government Department of Education (2017)				
Connected Beginnings [32]	Australian Government Department of Education (2016)				
A National Vision for Early Childhood Education and Care [33]	Australian Government Department of Education (2023^a^)				
National Mental Health and Suicide Prevention Plan [34]	Australian Government Department of Health and Aged Care (2021)				
National Mental Health and Wellbeing Pandemic Response Plan [35]	Australian Government Department of Health and Aged Care (2020)				
Australian Government response to contributing lives, thriving communities‐ review of mental health programmes and services [36]	Australian Government Department of Health and Aged Care (2015)				
The National Workforce Centre for Child Mental Health [37]	Australian Government Department of Health and Aged Care (2019)				
National Preventive Health Strategy 2021–2030 [38]	Australian Government Department of Health and Aged Care (2021)				
The new National Aboriginal and Torres Strait Islander Health Plan 2021–2031 [39]	Australian Government Department of Health and Aged Care (2021)				
Head to Health Kids National Service Model [40]	Australian Government Department of Health and Aged Care (2022)				
National Children's Mental Health and Wellbeing Strategy [11]	National Mental Health Commission (2021)				
Families and Children Activity Outcomes Framework [41]	Australian Government Department of Social Services (2021)				
Understanding and applying the Aboriginal and Torres Strait Islander Child Placement Principle: A resources for legislation, policy, and program development [42]	SNAICC‐ National Voice for our Children (2018)				
Aboriginal and Torres Strait Islander Child and Family Services Evaluation Readiness Toolkit [43]	SNAICC—National Voice for our Children (2019)				
Return to Country Framework [44]	Victorian Aboriginal Child Care Agency (2017)				
Framework to inform the development of a national Aboriginal and Torres Strait Islander early childhood strategy [45]	SNAICC—National Voice for our Children (2021)				
National Aboriginal and Torres Strait Islander early childhood strategy [46]	National Indigenous Australians Agency (2021)				
Early Years Strategy 2024–2034 [47]	Australian Government Department of Social Services and Australian Government Department of Education (2024)				

^a^ Draft policy published online for consultation.

Key: 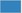, Yes; 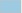, Yes, to some extent; 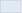, No.

### 3.1 Differentiation Between Child and Adolescent Mental Health

The majority of policies (*n* = 16) differentiated child mental health from the mental health of young people or adolescents, usually by specifically targeting children (e.g., Mental Health in Primary Schools) or by defining age ranges of interest (e.g., National Mental Health and Suicide Prevention Plan). Nine policies were identified as not differentiating between child and adolescent mental health at the overarching policy level; however, in the case of education‐based policies, it is likely they are differentiated at the implementation level, providing resources targeted towards primary schools (e.g., Framework for Improving Student Outcomes [FISO 2.0] and Mental Health Fund and Menu).

### 3.2 Promotion of Mental Health Competencies

Twenty policies aimed to target mental health competence, consistent with the broad nature of mental health competence, these competencies were defined differently across policies and targeted to different extents. Nine policies aimed to support the development of capabilities necessary for children to thrive and reach their full potential, one of the goals set out in A National Vision for Early Childhood Education and Care is, ‘All children are supported to reach their potential’. Five policies generally targeted wellbeing, including ensuring children have a strong sense of wellbeing, Outcome 3 of The Victorian early years learning and development framework is, ‘Children have a strong sense of wellbeing’. Social–emotional wellbeing was identified as a key target by three policies, Priority 6 of the National Aboriginal and Torres Strait Islander Health Plan 2021–2031 is ‘Social and emotional wellbeing and trauma‐aware, healing‐informed approaches’, additionally the priority specifies a focus on young people, ‘Social and emotional wellbeing approaches must focus on a strong start to life’. Finally, resilience was identified by two policies (e.g., The National Workforce Centre for Child Mental Health), and one measurement framework provided a competence indicator (e.g., Victorian child and adolescent outcome framework).

Of the Twenty policies that targeted competence, 10 included competence as a key aim or recommendation, usually accompanied by a clear definition of the competency being targeted:

Wellbeing means having good mental and physical health, including attachment, positive affect, and self‐regulation. This means being able to manage emotions productively and build resilience and persistence, being adaptable and confident, and experiencing feelings of satisfaction and happiness. Early childhood professionals, individually and together, play a key role with families in promoting healthy life practices and children's sense of wellbeing. (The Victorian early years learning and development framework)

A further four policies included explicit outcomes, activities or measures relating to competence, the Family and Children Activity Framework suggested outcome measures such as ‘positive mental health and wellbeing’, ‘increased resilience’ and ‘positive relationships’ to measure the aim ‘children and young people thrive’. Six policies targeted competence to a lesser extent, these policies identified competence as important but did not provide a clear definition of what was meant or it was not a key aim, the National Workforce Centre for Child Mental Health includes resilience as a guiding principle, but it is not central to the policy, and the draft National Vision for Early Childhood Education and Care aims to support children to reach their potential but does not define what is meant by potential.

Five policies did not target competence, instead focusing on prevention, early intervention and treatment of mental health difficulties. In some cases, these policies still referred to wellbeing but did not provide clear targets or aims. The National Preventive Health Strategy 2021–2030 references the Children's Mental Health and Wellbeing Strategy as a related strategic guide but did not provide specific mental health competence targets for children.

### 3.3 Prevention and Treatment of Mental Health Difficulties

Seventeen policies targeted mental health difficulties. Difficulties were targeted in one of three ways, the provision of mental health services for children, prevention and early intervention and through the definition of child mental health outcomes and indicators. As an example of service delivery, the Head to Health Kids National Service Model Hubs aim to ‘provide comprehensive, multidisciplinary care which supports children and their families’ which are ‘designed to operate as a secondary level child mental health and wellbeing service, targeting mild to moderate emerging complexity’. While the Mental Health Fund Menu also provides Tier 3 Targeted support, it's focus is prevention and early intervention through ‘Tier 1: Positive mental health promotion’ and ‘Tier 2: Early intervention and cohort specific support’. Finally, as an example of defining child mental heal outcomes and indicators, the Victorian child and adolescent outcome framework includes indicators to measure whether ‘children and young people are mentally and emotionally healthy’ such as ‘proportion of children on school entry with emotional and beahvioural difficulties’ and ‘rate of intentional self‐harm in young people’.

### 3.4 Supporting Families to Improve Child Mental Health Outcomes

Twelve policies aimed to support families as a way to improve child mental health outcomes. These policies identified specific strategies to leverage families to improve child outcomes, such as delivering parenting programs (Royal Commission, National Children's Mental Health and Wellbeing Strategy, Connected Beginnings), interventions and supports for attachment and relationships (Head to Health), cultural practices such as Birthing on Country (The new National Aboriginal and Torres Strait Islander Health Plan 2021–2031), and universal perinatal mental health screening (Prevention, Compassion, Care: National Mental Health and Suicide Prevention Plan). Seven out of 12 policies that specified strategies to support families were targeted at Aboriginal and Torres Strait Islander children and families, leaving only four universal policies that specified support for families.

Eleven policies acknowledged families as important for a child's mental health, measured family outcomes, provided family services and/or encouraged family engagement. Generally, these were less about leveraging family strengths to support child mental health and more about acknowledging the family as a key part of the ecological model; the National Preventive Health Strategy acknowledges:

Infants and children, more than any other age group, are shaped and influenced by a range of social, biological and environmental factors. Their mental health and wellbeing cannot be separated from the broader context of their lives, which includes their own individual characteristics, their family, school, local neighbourhood, work and community environments.

Despite this acknowledgement of the ecological model, the National Preventive Health Strategy does not provide details of how these contextual factors could be leveraged to promote and protect mental health. Four education, and one health policy identified family partnerships and engagement as key activities, these activities were not considered supporting families to influence child mental health, as they were more aligned with engaging families as stakeholders or acted as a moderator between the actions of the organisation and child outcomes. The FISO 2.0 *Engaging families in lifting student outcomes guide* defined engagement as building connectedness to school and promoting a sense of belonging for students and their families and saw families as a useful tool to provide valuable feedback and information to inform assessments of student wellbeing. While family engagement will likely influence child mental health outcomes, families are not specifically supported to improve child mental health outcomes in these policies. Two outcome frameworks identified family level indicators, such as parental confidence and self‐efficacy (Families and Children Activity Outcomes Framework), and healthy family functioning (Victorian child and adolescent outcome framework), however these were not specifically linked to child mental health outcomes.

Two education policies had no mention of families; these policies instead focused on school‐based interventions. However, it is likely that at the implementation level both the Mental Health Fund and Menu, and the Mental Health in Primary Schools programs engage families.

## 4 Discussion

We aimed to identify what current policy documents are being used in Victoria and nationally to inform work to improve child mental health outcomes. We described the extent infants and children are targeted separately from adolescents, and the extent to which these policies target competence and support families to influence child mental health. Overall, we found there is a broad range of policies, at a state and national level, that aim to prevent and treat mental health difficulties and promote mental health competence. However, without assessing the effectiveness of these policies in improving child mental health outcomes, it is not possible to determine if they are sufficient to address the mental health needs of children. Nonetheless, this breadth of policies demonstrates that policy makers recognise the importance of supporting the development of competencies and preventing and addressing difficulties, ensuring children have the best start to life.

Despite the breadth of policies observed, there was variability in the extent to which competence was targeted. We also identified a gap in the extent to which universal policies leverage and support families to drive changes in child mental health competence and difficulties; in contrast, Aboriginal and Torres Strait Islander policies were found to be family‐inclusive, a clear strength in the policy landscape.

### 4.1 Differentiation Between Child and Adolescent Mental Health

Most policies differentiated between childhood and adolescents; however, there were still some policies that did not clearly separate these two very distinct life stages. The lifecourse model describes health as dynamic, shaped by physical, biochemical, psychological, social, and cultural factors; it continuously develops over an individual's lifespan [48]. This development of health over the lifespan is also influenced by our age and life stage as we are exposed to different factors that influence our health at different ages. Children and adolescents experience different drivers of mental health [48, 49, 50, 51]. While there can be some overlap, the differing factors relevant to these different life stages mean it is inappropriate to target them together. To better target the key drivers of mental health outcomes, policies should clearly differentiate between children (0–12 years) and adolescents. Competence and mental health change over childhood [1]; it is possible that there are different factors influencing mental health outcomes at different timepoints; therefore, policies should also consider further differentiating between infancy and early childhood (0–5 years) and childhood (6–12 years).

### 4.2 Targeting Competence

While it is promising that most policies identified for inclusion in this review targeted competence, there was some variability in the extent this occurred. In particular, there was a failure by some policies to define what was meant by key competence outcomes such as wellbeing, thriving or full potential. The research and policy spaces are filled with overly broad definitions of wellbeing and can be made up of different wellbeing constructs [52, 53]. There have been efforts to clearly define wellbeing [52], and while many researchers now agree that it is made up of multiple constructs (e.g., social, emotional, physical), in practice, a universal definition or measurement does not exist [54]. This means policies cannot assume that references to wellbeing will be easily understood by those implementing them; a failure to explicitly define wellbeing (and other conceptualisations of positive mental health) will prevent accurate measurement and tracking of relevant outcomes. Conceptualising and measuring outcomes is key to understanding the effectiveness of policy implementation; a lack of information about outcomes can impede the ability to monitor and evaluate policy implementation [55, 56]. To ensure accurate decision making about the appropriateness and effectiveness of policies relating to child mental health competence, policymakers must first have a clear understanding of what to measure by defining competence.

### 4.3 Leveraging Families

This review showed that policies that target Aboriginal and Torres Strait Islander children and families are more likely to support families to improve child outcomes, compared to more general or universal policies. The National Aboriginal Community Controlled Health Organisation (NACCHO) defines health as ‘not just the physical well‐being of an individual but refers to the social, emotional and cultural well‐being of the whole Community in which each individual is able to achieve their full potential as a human being thereby bringing about the total well‐being of their Community’ [57]. Similarly, family/Community has been identified as a cultural determinant of health for Aboriginal and Torres Strait Islander people [58]. This strong interconnection between individual health outcomes and the health of families and Community may help explain why policies developed by Aboriginal and Torres Strait Islander peak bodies and government organisations are more likely to provide specific strategies to support families to achieve positive outcomes for children. While there was a gap in universal policies that identified specific strategies to support families, most policies acknowledged the importance of the family for a child's mental health outcomes. Often this was done by referencing or presenting an adapted ecological model [59].

A recent policy scoping review looked at how well the Australian primary health care system is able to identify and support families experiencing adversity [60]. The review found that there is currently political will and an authorising environment for policies that address family adversity, particularly through early intervention [60]. Policies identified in the review focused on a range of family adversities, some of which have well‐established impacts on child mental health outcomes, including parent mental illness [21, 61] and parenting [62, 63]. The current political will and authorising environment to address family adversity identified by Honisett et al., and the acknowledgement of families as a key factor in the mental health competence and difficulties of children identified in this review, show that supporting families is a viable strategy to support the mental health outcomes of children [60].

### 4.4 Multisectoral Approach to Targeting Child Mental Health

Most policies identified in the review were from across health and education departments, reflecting their established roles in promoting the health and development of children. This aligns with expectations, as these sectors have traditionally led efforts to promote mental health competence and address mental health difficulties in children. However, despite the role of social services departments in family support, early intervention and child protection, we only identified two relevant policies from the federal social service department and none from the Victorian social service department. Despite this gap, the recent release of the Early Years Strategy suggests a positive shift by the federal social services department towards promoting mental health competence through family‐focused policies within a strengths‐based lens [47].

Our review highlights a dominance of federal policies suggesting national leadership in addressing child mental health competence and difficulties. While we identified Victorian health and education policies that align with national efforts, the lack of state social service policies is surprising given state and territory governments are responsible for the care and protection of children experiencing and at risk of abuse and neglect [64]. The lack of policies explicitly targeting mental health competence suggests a missed opportunity to embed mental health promotion into broader family and child welfare initiatives. The divide between federal/state funding has been identified as a barrier to the mental health care of children [65], so ensuring these federal policies trickle down to influence state‐based systems will be important going forward. State‐based social services policies would be strengthened by considering and measuring their impact on child mental health outcomes; while they likely already have an impact on child mental health outcomes, without setting specific goals and monitoring impact we will be unable to know the extent this occurs.

The range of policies across departments and jurisdictions demonstrates that there is potential for a multisectoral response to child mental health, which has been identified as an important component in a public health response to child mental health [24]. To determine if this is a truly multisectoral response and not siloed efforts across departments, further research is needed to see if and how these policies are or can be coordinated across sectors. The work of the Early Years Strategy, which was launched by both the Minister for Social Services and the Minister for Early Childhood Education, is trying to achieve this [47]. Additionally, without assessing the efficacy of these policy responses, we are unable to determine if the current response is sufficient to improve the mental health competence of children. Future research could assess the quality and effectiveness of these policies.

## 5 Limitations

This policy scoping review presents an overview of the current policy landscape nationally and within Victoria, Australia, limiting its generalisability. However, as the majority of policies identified were federal policies, there is some indication of the overarching policy landscape in Australia. While not included in this review, there are relevant policies across all states and territories that aim to support children and families, such as the New South Wales Brighter Beginnings [66], Queensland's Putting Queensland Kids First [67], and the South Australian Royal Commission into Early Childhood Education and Care [15].

Finally, policies specific to other key priority populations such as children living in rural areas or children from migrant and refugee backgrounds did not emerge in the search of general policy websites. It may be that these policies have been developed by peak bodies, but more research is needed to confirm this.

## 6 Conclusion

This study aimed to understand the extent to which Victoria and federal policies target mental health competence; we found that there are a range of policies currently being implemented to improve child mental health by promoting competencies and preventing and addressing difficulties across different sectors both within Victoria and federally. While we identified a gap in policies that target competence from Victorian‐based social service departments, overall, the range of policies across departments and jurisdictions demonstrates that there is potential for a multisectoral response to child mental health. To assess the implementation fidelity, efficacy and coordination across sectors of this policy response would require additional research. We also explored whether policies explicitly targeted families as a key influence on child mental health competence and difficulties; policies that target Aboriginal and Torres Strait Islander children and families were identified as more likely to have a focus on families to improve child mental health outcomes; however, there was a gap in universal policies that identified specific strategies to support families. There is an opportunity to strengthen the current policy response by differentiating between children and adolescents, clearly defining competence outcomes, and by providing guidance in how policies and interventions can support families to improve child mental health outcomes.

## Ethics Statement

This is a policy scoping review and ethics approval was not required.

## Conflicts of Interest

The authors declare no conflicts of interest.

## Data Availability

Data sharing is not applicable to this article as no new data were created or analyzed in this study.

## Appendix A

**TABLE A1:** Policy scoping search terms, search location and search date.

Website	Date of search	Search terms	Filters applied
VictorianDepartment of Health	18.07.23	Child mental health and wellbeing	Community health; maternal child health; mental health; mental health and wellbeing reform; mental health services; population health systems; prevention and promotion; preventive health; public health; wellbeing and participation
Victorian Department of Education	18.07.23	Child mental health and wellbeing	n/a
Victorian Department of Families, Fairness and Housing	19.07.23	Child mental health and wellbeing; child wellbeing	n/a
Australian Government Department of Education	19.07.23	Child wellbeing; child mental health	n/a
Australian Government Department of Health and Aged Care	19.07.23	Child mental health and wellbeing	n/a
Australian Government Department of Social Services	27.07.23	Child mental health and wellbeing	n/a
Australian Policy Online	25.06.2024	Child wellbeing	n/a
